# Association between prognostic nutritional index and all-cause mortality among intestinal obstruction patients in the intensive care unit: a retrospective study

**DOI:** 10.3389/fnut.2025.1583201

**Published:** 2025-06-09

**Authors:** Yuanshuo Ge, Zhe Wang, Cheng Zhang

**Affiliations:** ^1^Department of General Surgery, General Hospital of Northern Theater Command (Formerly Called General Hospital of Shenyang Military Area), Shenyang, China; ^2^Jinzhou Medical University, Jinzhou, China

**Keywords:** prognostic nutritional index, intestinal obstruction, ICU, mortality, MIMIC-IV, nutritional status, critical care

## Abstract

**Background:**

Intestinal obstruction (IO) is a common surgical emergency associated with significant morbidity and mortality, particularly in critically ill ICU patients. The Prognostic Nutritional Index (PNI), calculated using serum albumin levels and total lymphocyte counts, has demonstrated prognostic value in various conditions. However, its role in critically ill IO patients remains unexplored.

**Methods:**

We conducted a retrospective cohort study using the MIMIC-IV database. Critically ill patients with IO were identified, and their PNI values on the first day of ICU admission were recorded. Patients were stratified into quartiles based on PNI and analyzed for 30-day, 60-day, and 90-day all-cause mortality. Multivariable Cox regression models adjusted for potential confounders, and restricted cubic splines examined the relationship between PNI and mortality risk.

**Results:**

A total of 701 patients were included in the analysis. Patients in the highest PNI quartile had significantly lower 30-day, 60-day, and 90-day all-cause mortality rates compared to those in the lowest quartile. After adjusting for covariates, higher PNI remained an independent predictor of reduced mortality (30-day HR 0.96, 95% CI: 0.93–0.98, *p* < 0.001; 60-day HR 0.96, 95% CI: 0.94–0.98, p < 0.001; 90-day HR 0.97, 95% CI: 0.95–0.99, *p* = 0.002).

**Conclusion:**

PNI is independently associated with lower mortality in critically ill IO patients, supporting its utility as a risk stratification tool in this population. These findings underscore the importance of early nutritional assessment and intervention, and highlight PNI’s potential to guide clinical decision-making in the ICU setting.

## 1 Introduction

Intestinal obstruction (IO) is a common surgical emergency, associated with high morbidity and healthcare costs (1). It involves either partial or complete blockage of the intestinal lumen, disrupting the normal gastrointestinal flow (2). This condition presents considerable risks, including bowel ischemia, perforation, sepsis, and an increased mortality rate among critically ill patients (3). Managing intestinal obstruction in the ICU is particularly challenging due to the compromised baseline health of these patients, which heightens their susceptibility to rapid clinical decline.

The Prognostic Nutritional Index (PNI), calculated using serum albumin levels and total lymphocyte counts, provides a quick and straightforward measure of a patient’s nutritional and immune status (4). PNI was initially developed as a preoperative risk assessment tool for surgical patients, but it has also shown prognostic value in various conditions such as cardiovascular diseases and chronic inflammatory disorders (5, 6). Furthermore, while many studies of IO mortality have focused on inflammatory markers such as red cell distribution width (RDW) (7), C-reactive protein (CRP) (8), and neutrophil-to-lymphocyte ratio (NLR) (9), there is a growing recognition of the critical role that nutritional status plays in influencing patient outcomes. By reflecting both protein reserves and immune competence, PNI offers an integrated assessment of a patient’s resilience, which is particularly crucial for those admitted to intensive care units, where metabolic stress and nutritional depletion can exacerbate the risk of adverse outcomes (10).

This study aims to evaluate the prognostic value of PNI in critically ill patients with IO using the MIMIC-IV database. By analyzing PNI values obtained on the first day of ICU admission, we aim to establish a reliable tool for early risk stratification in IO patients. Early identification of high-risk patients may allow clinicians to implement timely, targeted interventions that improve survival outcomes and optimize the use of ICU resources. This research underscores the critical role of incorporating nutritional and immune assessments into the management of IO and provides a foundation for future investigations that could inform clinical guidelines and practice standards.

## 2 Materials and methods

### 2.1 Research design

This research utilized the MIMIC-IV dataset (version 2.2), which consists of de-identified intensive care unit records from Beth Israel Deaconess Medical Center, spanning from 2008 to 2019. The dataset provides detailed information on patient demographics, laboratory test results, physiological indicators, therapeutic interventions, and clinical outcomes. To access the data, a data use agreement had to be completed, and certification in human research ethics was required. One of the authors (Ge ID: 13547277) met these conditions and carried out the data extraction and initial processing steps.

**Figure 1:**
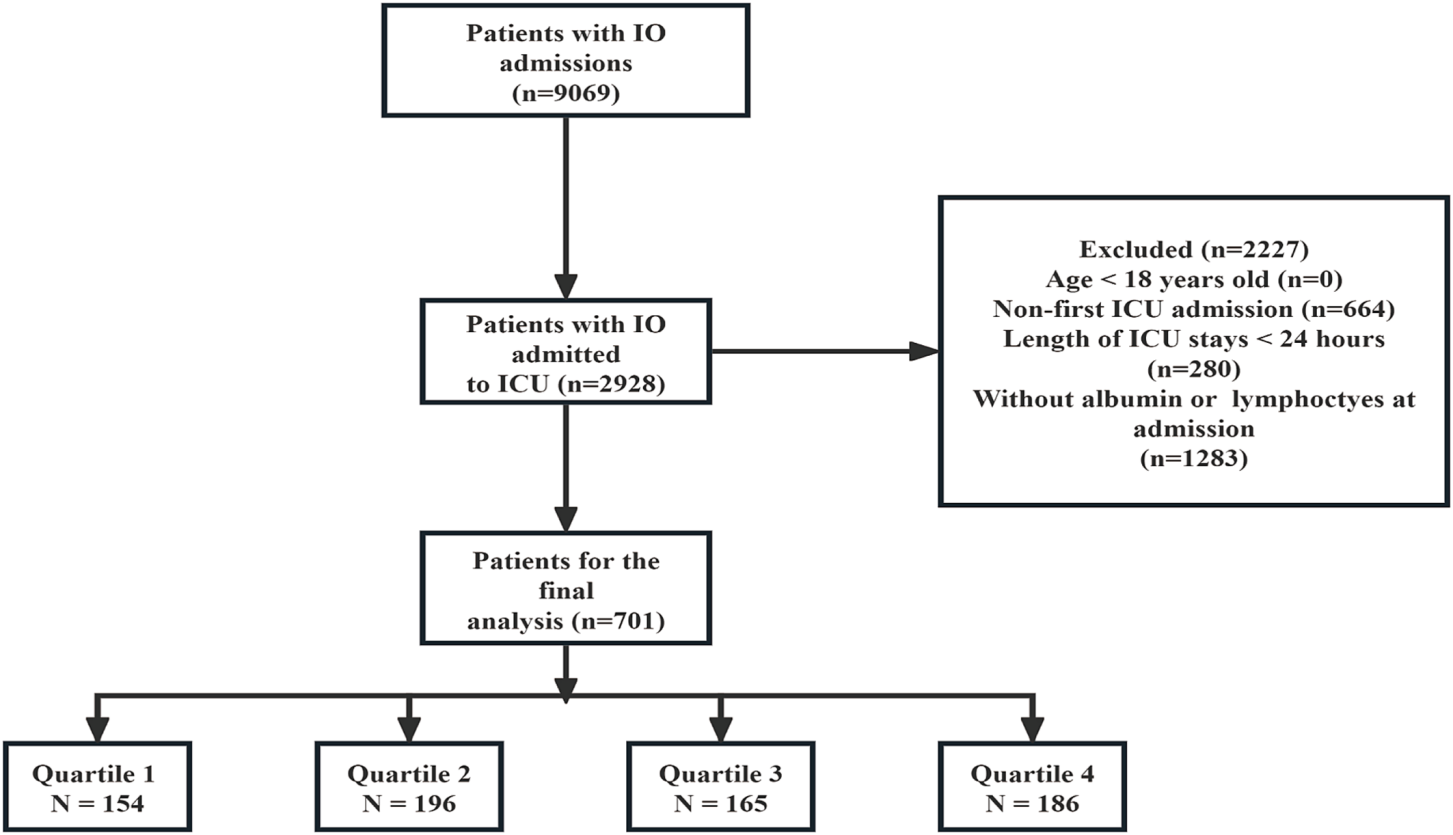
Patient flow through the trial. IO, Intestinal obstruction; ICU, Intensive Care Unit.

Initially, a total of 9,069 patients with IO admissions were identified. Among these, 2,928 patients were admitted to the ICU. After applying the exclusion criteria, 2,227 patients were excluded from the analysis. The exclusion criteria included: age less than 18 years (*n* = 0), non-first ICU admission (*n* = 664), ICU stays shorter than 24 h (*n* = 280), and patients without albumin or lymphocytes measured at admission (*n* = 1,283). This resulted in 701 patients being included in the final analysis. These patients were then stratified according to the quartiles of the PNI (Prognostic Nutritional Index) into four groups: Quartile 1 (*n* = 154), Quartile 2 (*n* = 196), Quartile 3 (*n* = 165), and Quartile 4 (*n* = 186) (Figure 1).

### 2.2 Data collection

Patient data were extracted from the MIMIC-IV (version 2.2) database using PostgreSQL, focusing on the first 24 h after ICU admission. Demographic variables included age, gender, race, weight, and insurance type. Clinical severity was measured by Charlson comorbidity index, Sequential Organ Failure Assessment (SOFA), and Acute Physiology Score III (APS III). Vital signs included heart rate, systolic blood pressure (SBP), respiratory rate, and oxygen saturation (SpO2). Comorbidities were recorded for diabetes, renal disease, malignant cancer, sepsis, and hypertension. Laboratory data included hemoglobin, white blood cell count (WBC), platelet count, albumin, lymphocytes, anion gap, sodium, potassium, international normalized ratio (INR), and total bilirubin. Treatment data included the use of mechanical ventilation, continuous renal replacement therapy (CRRT), and octreotide. Outcomes assessed were hospital length of stay, ICU length of stay, and hospital mortality at 30, 60, and 90 days.

### 2.3 Definition and clinical results

The primary outcome was all-cause mortality within 30 days after hospital admission, while secondary outcomes included all-cause mortality within 60 and 90 days after admission. The PNI is calculated using the formula: PNI = 10 × serum albumin level (g/dL) + 5 × total lymphocyte count (10⁹/L) (6). Both serum albumin and lymphocyte counts were obtained from routine venous blood samples analyzed by the hospital’s central clinical laboratory.

### 2.4 Statistical analysis

Continuous variables were summarized as mean ± SD or median based on distribution, and categorical variables as percentages. Normality was assessed using the Kolmogorov–Smirnov test. For normally distributed variables, t-tests or ANOVA were used for group comparisons; non-normally distributed variables were compared with the Mann–Whitney U test or Kruskal-Wallis test. Kaplan–Meier curves analyzed the association between PNI quartiles and 30-day, 60-day, and 90-day mortality, using log-rank tests for group comparisons. Variables with >20% missing data were excluded. For variables with ≤20% missingness, multiple imputation was performed using the multivariate imputation by chained equations method, implemented via the *mice* package in R, under the assumption of missing at random. Details of the variables with missing data and their respective proportions are provided in Supplementary Table S1. Cox proportional hazards models estimated HRs and 95% CIs for the associations between PNI and clinical outcomes, with PNI analyzed both as a continuous variable and in quartiles, using the lowest quartile as the reference and adjusting for relevant covariates. Confounders were selected based on univariate analysis (*p* < 0.05) and clinical relevance. Multivariable models included: Model 1 (unadjusted); Model 2 (adjusted for age, sex, race, and weight); and Model 3 (further adjusted for hemoglobin, anion gap, INR, total bilirubin, Charlson comorbidity index, SOFA, APSIII, and sepsis). To assess potential multicollinearity among covariates, we calculated variance inflation factors, including generalized VIF for categorical variables. A restricted cubic spline (RCS) regression model with three knots assessed the nonlinear relationship between baseline PNI and 30-, 60-, and 90-day mortality. To evaluate whether combining PNI with a commonly used disease severity score could improve prognostic performance, we conducted a joint analysis using the PNI and the SOFA, and calculated the area under the receiver operating characteristic (ROC) curve (AUC). In addition, the optimal cutoff value for the combined model was determined using the Youden Index. Interaction tests evaluated the prognostic impact of PNI across subgroups defined by sex, age (<65 and ≥65 years), cancer status, and sepsis. Robustness analyses were performed to assess the stability of our primary findings. First, the multivariable Cox regression was repeated using the original (non-imputed) dataset. Second, patients with ICU stays less than 24 h—excluded from the main analysis—were included and analyzed using the same covariate adjustments as Model 3. Both analyses were conducted for the primary outcome of 30-day all-cause mortality. In addition, to explore long-term prognostic value, we performed a separate Cox regression analysis for 360-day all-cause mortality. Statistical significance was defined as *p* < 0.05. All analyses were conducted using R software (version 4.4.2).

## 3 Results

### 3.1 Baseline characteristics

Table 1 presents the baseline characteristics of the cohort stratified by PNI quartiles. Quartile 4 had a significantly higher proportion of male patients (*p* = 0.010). SOFA and APS III scores were lower in Quartile 4 compared to Quartile 1 (*p* < 0.001 for both). Heart rate decreased while SBP increased in Quartile 4 (*p* < 0.001 for both). The occurrence of malignant cancer and sepsis was lower in Quartile 4 (*p* = 0.001 and *p* = 0.019, respectively). Laboratory results showed higher albumin, lymphocyte counts, and total bilirubin levels (*p* < 0.001, *p* = 0.005, *p* = 0.009, respectively) as well as lower INR values in Quartile 4 (*p* = 0.004). Mortality rates at 30, 60, and 90 days were significantly reduced in Quartile 4 compared to Quartile 1 (*p* < 0.001 for all).

**Table 1:** Baseline characteristics stratified by PNI quartiles.

Characteristic	Overall *N* = 701	Quartile 1 *N* = 154	Quartile 2 *N* = 196	Quartile 3 *N* = 165	Quartile 4 *N* = 186	*p*-value
Demographics						
Age (years)	64.15 (53.45, 73.75)	63.16 (52.19, 72.73)	64.43 (54.97, 74.02)	64.51 (54.01, 74.61)	63.26 (50.89, 73.26)	0.661
Gender, male (%)	447 (64%)	86 (56%)	119 (61%)	107 (65%)	135 (73%)	**0.010**
Race, white (%)	484 (69%)	107 (69%)	142 (72%)	114 (69%)	121 (65%)	0.482
weight (Kg)	80.00 (67.90, 97.40)	78.20 (66.20, 96.30)	78.15 (68.00, 97.35)	82.50 (67.00, 97.70)	83.40 (68.60, 98.00)	0.634
Insurance, *n* (%)						0.732
Medicaid	130 (19%)	26 (17%)	38 (19%)	31 (19%)	35 (19%)	
Medicare	363 (52%)	75 (49%)	97 (49%)	93 (56%)	98 (53%)	
Other	32 (5%)	8 (5%)	7 (4%)	6 (4%)	11 (6%)	
Private	176 (25%)	45 (29%)	54 (28%)	35 (21%)	42 (23%)	
Clinical scores						
Charlson comorbidity index	5.00 (3.00, 7.00)	5.00 (3.00, 8.00)	5.00 (3.00, 7.00)	5.00 (3.00, 8.00)	5.00 (3.00, 7.00)	0.144
SOFA	7.00 (4.00, 10.00)	8.00 (5.00, 11.00)	7.00 (5.00, 11.00)	6.00 (3.00, 9.00)	5.50 (3.00, 9.00)	**<0.001**
APS III	57.00 (44.00, 76.00)	71.00 (56.00, 88.00)	59.00 (44.00, 73.00)	51.00 (41.00, 69.00)	50.00 (37.00, 65.00)	**<0.001**
Vital signs						
Heart rate (bpm)	78.00 (67.00, 91.00)	84.00 (72.00, 95.00)	78.00 (69.50, 89.50)	79.00 (68.00, 93.00)	73.00 (64.00, 87.00)	**<0.001**
SBP (mmHg)	85.00 (77.00, 96.00)	83.00 (73.00, 90.00)	83.50 (76.00, 92.00)	86.00 (78.00, 98.00)	90.00 (80.00, 101.00)	**<0.001**
Respiratory rate (bpm)	13.00 (10.00, 16.00)	13.00 (10.00, 16.00)	12.00 (10.00, 16.00)	14.00 (11.00, 16.00)	13.00 (10.00, 15.00)	0.062
SpO2 (%)	96.92 (95.38, 98.41)	97.11 (95.50, 98.12)	97.02 (95.27, 98.53)	96.67 (95.12, 98.44)	96.90 (95.57, 98.64)	0.721
Comorbidities (%)						
Diabetes, *n* (%)	183 (26%)	35 (23%)	51 (26%)	51 (31%)	46 (25%)	0.381
Renal Disease, *n* (%)	144 (21%)	31 (20%)	32 (16%)	36 (22%)	45 (24%)	0.280
Malignant Cancer, *n* (%)	142 (20%)	40 (26%)	50 (26%)	31 (19%)	21 (11%)	0.001
Sepsis, *n* (%)	573 (82%)	131 (85%)	171 (87%)	128 (78%)	143 (77%)	**0.019**
Hypertension, *n* (%)	234 (33%)	53 (34%)	67 (34%)	47 (28%)	67 (36%)	0.477
Laboratory test						
Hemoglobin (g/L)	9.40 (7.90, 11.00)	8.60 (7.30, 10.00)	8.95 (7.85, 10.10)	9.80 (8.40, 11.50)	10.50 (8.80, 11.90)	**<0.001**
Platelets (10^9^/L)	165.00 (101.00, 251.00)	151.50 (72.00, 249.00)	155.50 (87.00, 266.00)	188.00 (119.00, 269.00)	167.00 (117.00, 223.00)	0.063
WBC (10^9^/L)	9.60 (6.20, 14.50)	11.15 (6.90, 16.40)	9.70 (5.80, 15.30)	9.40 (6.00, 12.80)	9.20 (6.40, 12.20)	0.062
Albumin (g/dL)	2.80 (2.40, 3.30)	2.05 (1.90, 2.20)	2.60 (2.50, 2.70)	3.00 (2.90, 3.10)	3.65 (3.40, 3.90)	**<0.001**
Lymphocytes (10^9^/L)	0.77 (0.46, 1.31)	0.74 (0.43, 1.18)	0.70 (0.41, 1.21)	0.86 (0.49, 1.35)	0.87 (0.56, 1.48)	**0.005**
PNI	28.02 (24.00, 33.00)	20.51 (19.00, 22.01)	26.00 (25.00, 27.01)	30.01 (29.01, 31.01)	36.51 (34.01, 39.01)	**<0.001**
Anion gap (m q/L)	13.00 (11.00, 16.00)	13.00 (10.00, 15.00)	13.00 (10.00, 16.00)	13.00 (11.00, 16.00)	14.00 (11.00, 16.00)	0.132
Sodium (mmol/L)	136.00 (132.00, 139.00)	136.00 (132.00, 138.00)	135.00 (132.00, 139.00)	137.00 (133.00, 140.00)	136.00 (131.00, 139.00)	**0.113**
Potassium (mmol/L)	3.80 (3.40, 4.20)	3.75 (3.40, 4.20)	3.80 (3.40, 4.20)	3.90 (3.50, 4.30)	3.80 (3.40, 4.20)	0.153
INR	1.30 (1.10, 1.60)	1.30 (1.20, 1.60)	1.40 (1.20, 1.70)	1.30 (1.10, 1.50)	1.20 (1.10, 1.50)	**0.004**
Total Bilirubin (umol/L)	0.80 (0.40, 1.90)	0.95 (0.40, 2.10)	1.00 (0.50, 2.60)	0.70 (0.40, 1.80)	0.60 (0.40, 1.30)	**0.009**
Treatments						
Mechanical Ventilation, *n* (%)	435 (62%)	103 (67%)	127 (65%)	95 (58%)	110 (59%)	0.238
CRRT, *n* (%)	75 (11%)	21 (14%)	17 (9%)	21 (13%)	16 (9%)	0.284
Octreotide, *n* (%)	75 (11%)	17 (11%)	24 (12%)	16 (10%)	18 (10%)	0.829
Events						
Los of Hospital (day)	15.59 (9.17, 24.80)	17.66 (8.94, 28.05)	15.68 (9.36, 27.40)	14.62 (9.98, 22.75)	14.46 (8.48, 22.18)	0.296
Los of ICU (day)	4.15 (2.18, 9.75)	4.30 (2.07, 10.14)	4.30 (2.30, 9.30)	4.01 (2.23, 9.05)	4.16 (1.99, 10.85)	0.965
30-day hospital Mortality (%)	183 (26%)	58 (38%)	53 (27%)	41 (25%)	31 (17%)	**<0.001**
60-day hospital Mortality (%)	233 (33%)	71 (46%)	70 (36%)	50 (30%)	42 (23%)	**<0.001**
90-day hospital Mortality (%)	255 (36%)	73 (47%)	78 (40%)	53 (32%)	51 (27%)	**<0.001**
360-day hospital Mortality (%)	289 (41.23)	78 (50.65)	87 (44.39)	62 (37.58)	62 (33.33)	**0.007**

PNI, Quartile 1 (9.00–24.00), Quartile 2 (24.00–28.02), Quartile 3 (28.02–33.00), Quartile 4 (33.00–55.01). PNI, prognostic nutritional index; SBP, Systolic blood pressure; WBC, White blood cell count; RBC, Red blood cell count; Platelet, Platelet count; SOFA, Sequential organ failure assessment; APS III, Acute Physiology Score III; SpO2, Oxygen saturation; INR, International normalized ratio; CRRT, Continuous renal replacement therapy. The variables with bold *p*-values are statistically significant.

### 3.2 Kaplan–Meier survival curve

**Figure 2:**
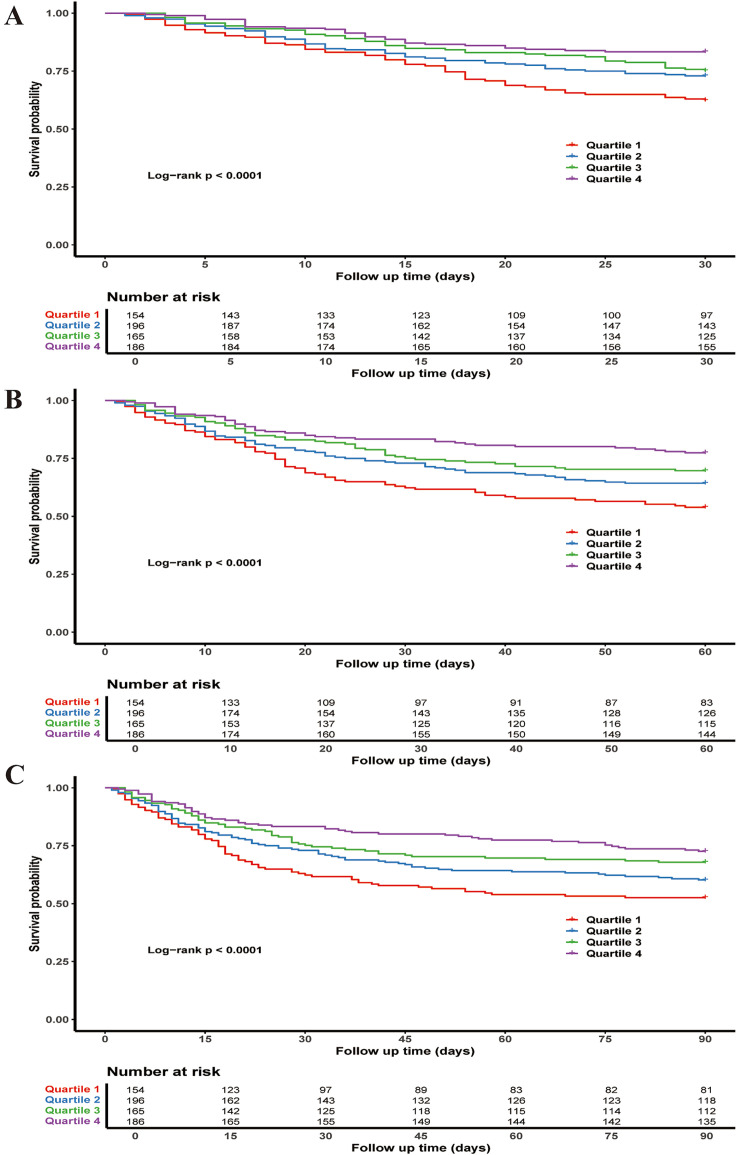
Kaplan–Meier survival curves for all-cause mortality: **(A)** 30-day, **(B)** 60-day, and **(C)** 90-day.

The Kaplan–Meier curves for 30, 60, and 90 days show statistically significant differences in survival probabilities between the PNI quartiles (log-rank *p*-values < 0.0001 at all time points). The fourth quartile consistently exhibits the highest survival probability across all follow-up periods (Figure 2).

### 3.3 Association between PNI and risk of mortality

**Figure 3:**
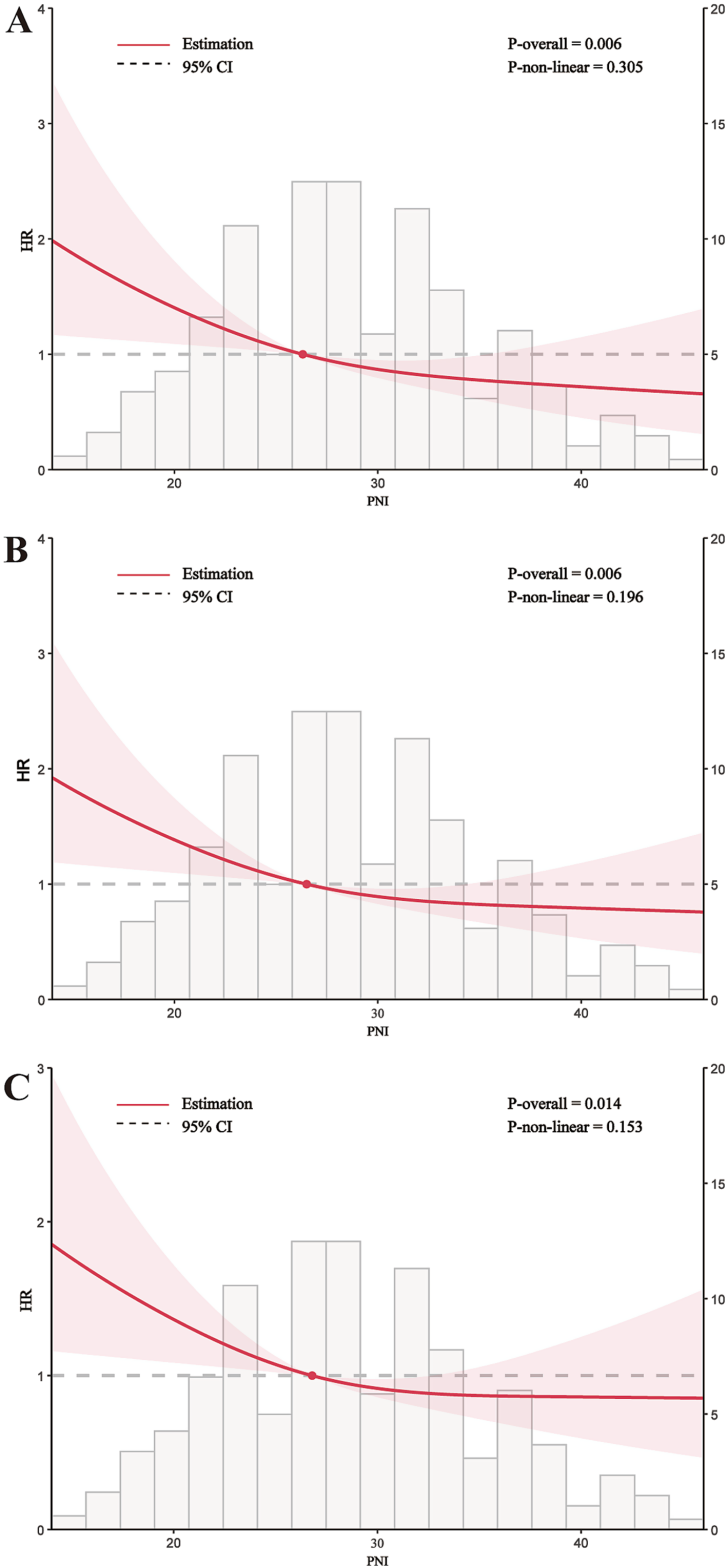
Restricted cubic spline curves for PNI hazard ratio. The central lines represent the adjusted hazard ratios, while the shaded ribbons indicate the 95% confidence interval (CI). **(A)** 30-day, **(B)** 60-day, and **(C)** 90-day all-cause mortality. HR, hazard ratio; CI, confidence interval; PNI, Prognostic Nutritional Index.

The variables included in the multivariable Cox regression (Table 2) were selected based on univariable Cox regression analysis (Supplementary Table S2) and recommendations from clinical experts. In the fully adjusted model (Model 3), higher PNI as a continuous variable was associated with lower all-cause mortality risk at 30 days (HR 0.96, 95% CI: 0.93–0.98, *p* < 0.001), 60 days (HR 0.96, 95% CI: 0.94–0.98, p < 0.001), and 90 days (HR 0.97, 95% CI: 0.95–0.99, *p* = 0.002). When analyzed as a categorical variable, Quartile 4 of PNI showed significantly reduced mortality risk compared to Quartile 1 at 30 days (HR 0.47, 95% CI: 0.29–0.74, *p* = 0.001), 60 days (HR 0.54, 95% CI: 0.26–0.57, *p* = 0.003), and 90 days (HR 0.61, 95% CI: 0.42–0.90, *p* = 0.012). Trend tests for PNI quartiles were statistically significant at all three time points (p = 0.001, *p* = 0.002, and *p* = 0.005, respectively). RCS regression indicated a linear decrease in 30-, 60-, and 90-day mortality risk with increasing PNI (non-linearity *p*-values: 30-day 0.305, 60-day 0.196, 90-day 0.153) (Figure 3). To ensure model stability, multicollinearity among covariates was assessed using variance inflation factors. All adjusted GVIF values were <2, suggesting no significant multicollinearity in the fully adjusted model (Supplementary Table S3). For the primary outcome of 30-day all-cause mortality, the AUC for SOFA alone was 0.68, while the combined SOFA/PNI model yielded an AUC of 0.71. The optimal cutoff value for the SOFA/PNI model was 0.34 (Supplementary Table S4).

**Table 2:** Multivariable cox regression analysis of PNI and all-cause mortality.

Variables	Model 1		Model 2		Model 3	
	HR (95%CI)	*p*	HR (95%CI)	*p*	HR (95%CI)	*p*
30-day						
Continuous variable	0.96 (0.94, 0.98)	**<0.001**	0.95 (0.93, 0.97)	**<0.001**	0.96 (0.93, 0.98)	**<0.001**
PNI quartiles						
Quartile 1	—		—		—	
Quartile 2	0.68 (0.47, 0.99)	**0.042**	0.64 (0.44, 0.93)	**0.020**	0.70 (0.47, 1.02)	0.066
Quartile 3	0.60 (0.40, 0.90)	**0.013**	0.55 (0.23, 0.55)	**0.004**	0.58 (0.38, 0.89)	**0.013**
Quartile 4	0.39 (0.25, 0.61)	**<0.001**	0.36 (0.23, 0.55)	**<0.001**	0.47 (0.29, 0.74)	**0.001**
P for trend		**<0.001**		**<0.001**		**0.001**
60-day						
Continuous variable	0.96 (0.94, 0.98)	**<0.001**	0.95 (0.93, 0.97)	**<0.001**	0.96 (0.94, 0.98)	**<0.001**
PNI quartiles						
Quartile 1	—		—		—	
Quartile 2	0.72 (0.52, 1.00)	0.051	0.68 (0.49, 0.95)	**0.025**	0.77 (0.55, 1.09)	0.139
Quartile 3	0.58 (0.41, 0.84)	**0.004**	0.55 (0.38, 0.79)	**0.001**	0.62 (0.42, 0.92)	**0.017**
Quartile 4	0.42 (0.28, 0.61)	**<0.001**	0.39 (0.26, 0.57)	**<0.001**	0.54 (0.26, 0.57)	**0.003**
P for trend		**<0.001**		**<0.001**		**0.002**
90-day						
Continuous variable	0.96 (0.94, 0.98)	**<0.001**	0.96 (0.94, 0.97)	**<0.001**	0.97 (0.95, 0.99)	**0.002**
PNI quartiles						
Quartile 1	—		—		—	
Quartile 2	0.77 (0.56, 1.07)	0.117	0.74 (0.54, 1.02)	0.062	0.82 (0.59, 1.15)	0.254
Quartile 3	0.60 (0.42, 0.85)	**0.004**	0.56 (0.39, 0.79)	**0.001**	0.62 (0.43, 0.91)	**0.015**
Quartile 4	0.48 (0.34, 0.69)	**<0.001**	0.45 (0.31, 0.64)	**<0.001**	0.61 (0.42, 0.90)	**0.012**
P for trend		**<0.001**		**<0.001**		**0.005**

Model 1: Crude. Model 2: Adjusted for Age, Gender, Race, Insurance. Model 3: Adjusted for Model2 + SOFA + Charlson comorbidity index + APS III + Hemoglobin, Anion gap, INR, Total Bilirubin, and Sepsis. PNI, Quartile 1 (9.00–24.00), Quartile 2 (24.00–28.02), Quartile 3 (28.02–33.00), Quartile 4 (33.00–55.01). PNI, prognostic nutritional index; SOFA, Sequential organ failure assessment; APS III, Acute Physiology Score III; INR, International normalized ratio. The variables with bold *p*-values are statistically significant.

### 3.4 Subgroup analysis

**Figure 4:**
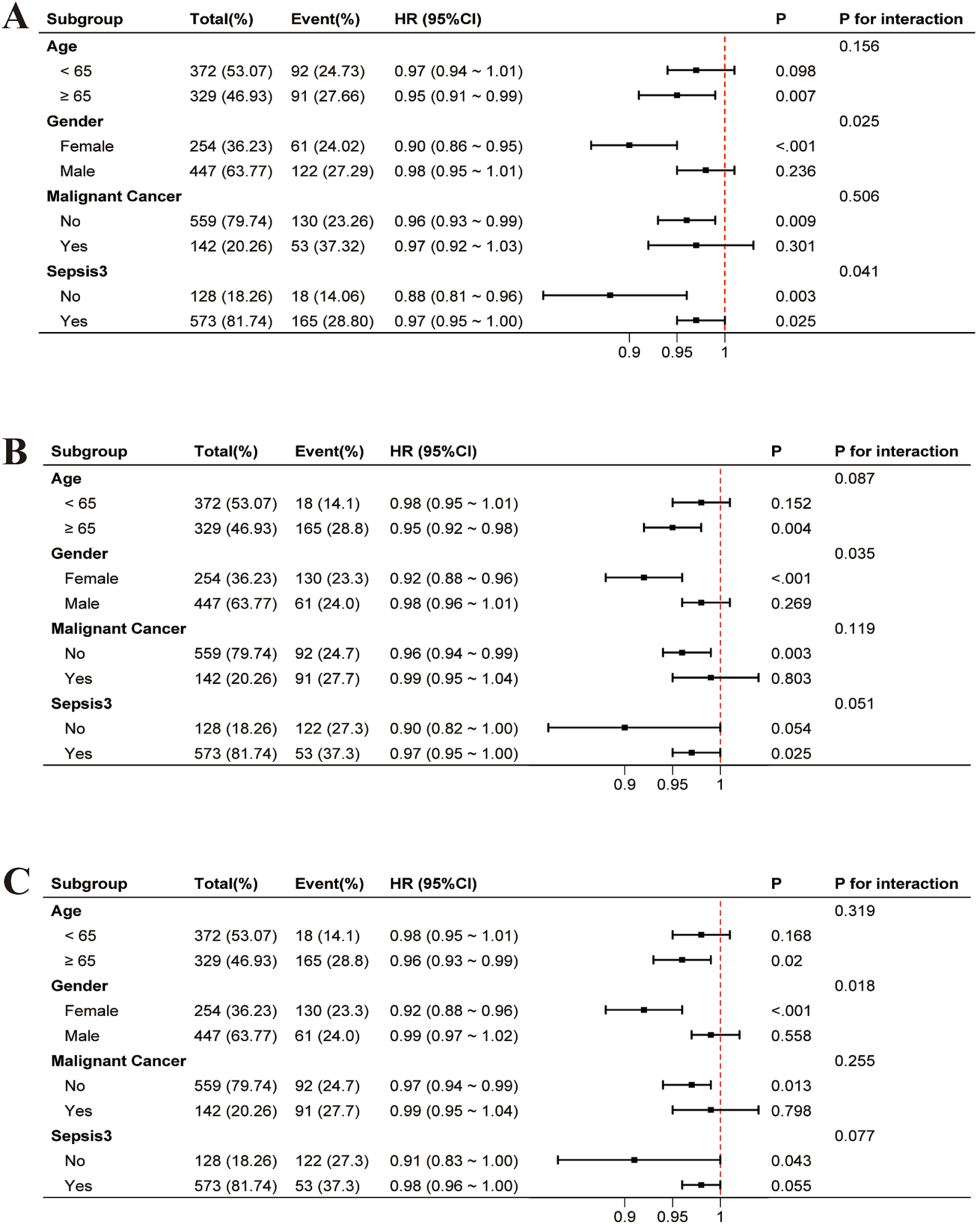
Adjusted forest plots of HR for all-cause mortality by subgroup, accounting for covariates: **(A)** 30-day, **(B)** 60-day, and **(C)** 90-day. HR, hazard ratio; CI, confidence interval.

After adjusting for covariates, interaction tests were conducted in predefined subgroups (Figure 4). Significant interaction effects were observed for gender (*p* = 0.025 at 30 days, *p* = 0.035 at 60 days, *p* = 0.018 at 90 days) and sepsis (*p* = 0.041 at 30 days) (Figure 4).

### 3.5 Robustness analyses

Sensitivity analyses showed that the association between higher PNI and lower 30-day all-cause mortality remained consistent in both the non-imputed dataset (HR = 0.96, 95% CI: 0.93–0.98; *p* < 0.001) and the full ICU cohort including patients with stays <24 h (HR = 0.96, 95% CI: 0.94–0.98; *p* < 0.001). In addition, PNI remained significantly associated with 360-day mortality (HR = 0.98, 95% CI: 0.96–0.99; *p* = 0.037) (Supplementary Table S5).

## 4 Discussion

This study explored the association between PNI and clinical outcomes in critically ill ICU patients with IO. Results demonstrated that patients with lower PNI had significantly higher 30-day, 60-day, and 90-day all-cause mortality rates compared to those with higher PNI. After adjusting for potential confounders, higher PNI remained consistently associated with lower mortality across these timeframes, supporting PNI as an independent risk factor. Therefore, PNI may serve as a valuable tool for assessing nutritional status and predicting mortality risk in critically ill patients with IO.

Previous studies on mortality in IO patients have often focused on inflammatory markers, such as RDW (7), C-reactive protein (8), and NLR (9). While these markers provide valuable insights into the systemic inflammatory response, they have notable limitations. Primarily, they do not reflect the patient’s overall nutritional status or long-term immune competence. In contrast, PNI has been investigated in various disease contexts—ranging from gastrointestinal malignancies to cardiovascular conditions—and has consistently demonstrated prognostic utility (11, 12). Building on these findings, our study extends the application of PNI to a critically ill ICU cohort with IO, a group particularly vulnerable to nutritional and metabolic derangements. By taking advantage of PNI’s simplicity, rapid risk stratification can be performed on the first day of ICU admission. This novel focus addresses a critical gap in existing research and highlights the importance of incorporating nutritional assessments into the management of IO patients admitted to the ICU.

The pathophysiological characteristics of IO—such as prolonged fasting, increased intraluminal pressure (13), bacterial translocation, and systemic inflammatory responses—directly impact albumin levels and lymphocyte counts, which are critical components of the PNI. Given that both hypoalbuminemia and lymphopenia are independently linked to adverse clinical outcomes (14, 15), the prognostic utility of PNI likely stems from the integrated contributions of its two components. Albumin, a negative acute-phase reactant, decreases not only due to reduced nutritional intake caused by fasting but also because of heightened inflammatory processes inherent to obstruction (16, 17). The increased capillary permeability, cytokine release, and hepatic reprioritization of protein synthesis during systemic inflammation lead to a decline in circulating albumin levels (18). Hypoalbuminemia subsequently disrupts oncotic pressure, contributing to fluid shifts, tissue edema, and impaired perfusion, which ultimately compromise organ function and healing (19). Lymphocyte counts, another key element of the PNI, are suppressed both by malnutrition and the systemic inflammatory environment (20). Prolonged fasting and poor caloric intake diminish lymphopoiesis, while ongoing inflammation induces lymphocyte apoptosis and shifts immune cell populations toward a myeloid-dominant response (21). The lymphocyte depletion weakens cellular immunity, rendering patients more susceptible to infections and sepsis (22). In the context of intestinal obstruction, this loss of immune competence is particularly concerning because bacterial translocation from the compromised gut barrier further amplifies systemic inflammation and the risk of secondary infections (23). Together, these processes form a feedback loop: intestinal obstruction drives systemic inflammation, which further depletes albumin and lymphocytes (24). The resulting hypoalbuminemia and lymphopenia weaken healing capacity, reduce immune defense, and exacerbate organ dysfunction, ultimately leading to disease progression and a significantly increased risk of mortality in patients with intestinal obstruction (25).

In the subgroup analysis, the protective effect of PNI on mortality was more pronounced in females and patients without sepsis. A possible mechanism is that females often have higher levels of certain immune cells, such as dendritic cells and T-helper cells, which play a key role in immune responses to infections and tissue damage (26). These differences in immune function may allow females to more effectively combat illness, promoting better recovery when nutritional status is optimized (27). Additionally, there are metabolic differences between males and females that may influence how they utilize nutrients and recover from illness. For example, females may have more efficient mitochondrial function, which is crucial for energy production and immune function, further aiding their recovery when nutritional status is optimized. Nevertheless, we acknowledge the possibility of residual confounding, including unmeasured hormonal or inflammatory factors, and these findings should be further explored in future studies with biomarker-based stratification. In contrast, immune dysregulation and heightened systemic inflammation in septic patients likely diminish the predictive utility of PNI. In patients without sepsis, the absence of excessive inflammation allows PNI to better reflect baseline nutritional and immune reserves, making it a more reliable predictor of mortality. Similarly, PNI did not reach statistical significance in patients younger than 65 years and those with malignant tumors. A possible explanation is that younger patients may have better nutritional reserves, which could diminish the observable impact of PNI on mortality, as they often receive more aggressive interventions and have better recovery potential. In patients with malignant tumors, cancer-related systemic inflammation and cachexia often dominate the clinical picture, overshadowing the role of PNI, while tumor-specific factors, such as tumor burden and treatment response, may play a more significant role in determining mortality risk.

Our findings have important clinical implications for the management of critically ill IO patients. Integrating PNI into the standard risk assessment protocol at ICU admission allows clinicians to quickly identify patients with a poor nutritional and immune profile who are at elevated risk of adverse outcomes. Early recognition of these high-risk patients provides a critical window for timely interventions, including individualized nutritional supplementation (such as albumin infusion), more aggressive management of fluid and electrolyte imbalances, and targeted monitoring of organ function. In addition, leveraging PNI as a prognostic tool can help guide decisions about resource allocation—such as prioritizing the use of advanced imaging modalities or more frequent laboratory testing—to ensure that the most vulnerable patients receive the appropriate level of care. Nevertheless, in low-resource ICU settings, delayed or unavailable albumin and lymphocyte measurements may limit the real-time applicability of PNI, which should be considered in future implementation. Beyond short-term outcomes, the routine use of PNI can inform the development of more personalized, evidence-based treatment pathways that optimize long-term survival and recovery for IO patients in the ICU setting. By shifting the focus to an integrated assessment of both inflammatory and nutritional parameters, our study addresses a crucial gap in the current standard of care and paves the way for more comprehensive risk stratification and intervention strategies in this high-risk population.

This study has several limitations. First, the retrospective design of this study may introduce selection bias. Although we adjusted for multiple confounding variables, residual confounding cannot be entirely excluded due to the limited inclusion of certain clinical factors or interventions, such as surgical treatments. However, the primary aim of this study was to rapidly assess nutritional status and perform early risk stratification based on PNI at ICU admission, and our analysis remains clinically informative within this context. Moreover, as an observational study, this research demonstrates association rather than causation. Further prospective cohort studies and interventional trials are needed to elucidate the causal relationship between PNI and mortality. Second, we excluded patients without albumin or lymphocyte measurements, which may have introduced selection bias and, to some extent, limited the generalizability of the findings. Third, this study assessed only the PNI value at ICU admission for the purpose of early risk stratification in critically ill IO patients. However, given that nutritional and immune status may fluctuate significantly throughout the ICU stay, the absence of serial PNI measurements limited our ability to evaluate its temporal trends and prognostic value. In addition, due to substantial missingness of CRP data and the absence of IL-6 measurements in our dataset, we were unable to validate the proposed inflammation-related mechanisms underlying hypoalbuminemia and lymphopenia. Additionally, relying solely on PNI as a primary nutritional marker may not capture more dynamic or comprehensive aspects of nutritional status, such as body composition or micronutrient levels. Although this study evaluated survival at multiple time points, long-term functional status could not be assessed due to data limitations, which should be explored in future research. Lastly, the generalizability of our findings is restricted, as the patient cohort from the MIMIC-IV database may not be representative of more diverse populations across different healthcare systems or regions. Future studies should focus on large-scale, multicenter, prospective cohorts across diverse racial and ethnic populations to further validate our findings and refine risk stratification methods.

## 5 Conclusion

In this study, we demonstrated that a higher PNI is independently associated with lower mortality in critically ill ICU patients with IO, even after adjusting for potential confounders. These findings suggest that PNI provides a valuable measure of both nutritional and immune status, offering an integrated alternative to traditional inflammatory markers and highlighting the importance of nutritional assessments in this high-risk population. By facilitating early risk stratification and guiding targeted interventions, PNI has the potential to improve patient outcomes and optimize resource allocation in the ICU setting. However, while our results underscore the promise of PNI as a prognostic tool, further prospective studies are needed to confirm its utility and clarify its role in clinical decision-making for critically ill IO patients.

## Data Availability

Publicly available datasets were analyzed in this study. This data can be found at: All data and materials are accessible at https://mimic.mit.edu/.
