# Supplementary material for: Association between prognostic nutritional index and all-cause mortality among intestinal obstruction patients in the intensive care unit: a retrospective study
Source: Front Nutr. 2025 Jun 9;12:1583201. doi: 10.3389/fnut.2025.1583201 (PMC12183055; doi:10.3389/fnut.2025.1583201)
Supplement: Supplementary file 1 [file Supplementary_file_1.DOCX]

***Supplementary Material***

**Table S1 Missing values for variables**

**Table S2 Univariate Cox proportional hazard ratios (HR) for 30-day all-cause mortality in patients.**

**Table S3 variance inflation factors (VIFs) for covariates included in the multivariable model**

**Table S4 discriminative performance of sofa and combined SOFA/PNI models for predicting 30-day all-cause mortality**

**Table S5 sensitivity analyses of the association between PNI and all-cause mortality**

**Table S1：Missing values for variables**

| **Variables** | **%** |
| --- | --- |
| INR | 3.138 |
| Bilirubin Total | 3.281 |
| Resp Rate(bpm) | 0.143 |
| Heart Rate(bpm) | 0.143 |
| Spo2 | 0.143 |
| Weight | 4.422 |

**Table S2：Univariate Cox proportional hazard ratios (HR) for 30-day all-cause mortality in patients.**

| **Variables** | **P** | **HR (95%CI)** |
| --- | --- | --- |
| 30-day mortality |  |  |
| Gender |  |  |
| female |  | 1.00 (Reference) |
| male | 0.296 | 1.18 (0.87 ~ 1.60) |
| Race |  |  |
| white |  | 1.00 (Reference) |
| black | 0.616 | 1.14 (0.69 ~ 1.87) |
| other | 0.008 | 1.56 (1.12 ~ 2.17) |
| Age | 0.015 | 1.01 (1.01 ~ 1.02) |
| Weight | 0.915 | 1.00 (0.99 ~ 1.01) |
| APS Ⅲ | <.001 | 1.03 (1.02 ~ 1.03) |
| Charlson Comorbidity Index | <.001 | 1.14 (1.09 ~ 1.20) |
| Sofa | <.001 | 1.15 (1.12 ~ 1.19) |
| Bilirubin Total | <.001 | 1.07 (1.05 ~ 1.09) |
| Anion gap | <.001 | 1.12 (1.08 ~ 1.15) |
| Sepsis |  |  |
| No |  | 1.00 (Reference) |
| Yes | <.001 | 2.31 (1.42 ~ 3.75) |
| Anion gap | <.001 | 1.12 (1.08 ~ 1.15) |
| Hemoglobin | 0.034 | 0.93 (0.87 ~ 0.99) |
| INR | <.001 | 1.36 (1.24 ~ 1.48) |

**Table S3 variance inflation factors (VIFs) for covariates included in the multivariable model**

| **Variable** | **GVIF** | **GVIF^(1/(2*Df))** |
| --- | --- | --- |
| Gender | 1.086365 | 1.042289 |
| Race | 1.053835 | 1.013195 |
| Weight | 1.101598 | 1.04957 |
| Hemoglobin | 1.126192 | 1.061222 |
| Anion gap | 1.221404 | 1.105171 |
| INR | 1.145184 | 1.070132 |
| Bilirubin total | 1.282851 | 1.13263 |
| Charlson comorbidity index | 1.104646 | 1.051021 |
| SOFA | 2.544573 | 1.595172 |
| APS Ⅲ | 2.233481 | 1.494484 |
| Sepsis3 | 1.175678 | 1.084287 |

**Table S4 discriminative performance of sofa and combined SOFA/PNI models for predicting 30-day all-cause mortality**

| **Variable** | **AUC (95%CI)** | **Accuracy (95%CI)** | **Sensitivity (95%CI)** | **Specificity (95%CI)** | **PPV (95%CI)** | **NPV (95%CI)** | **Cut off** |
| --- | --- | --- | --- | --- | --- | --- | --- |
| SOFA/PNI | 0.71 (0.66-0.75) | 0.71 (0.68-0.74) | 0.77 (0.74 - 0.81) | 0.53 (0.46 - 0.60) | 0.82 (0.79 - 0.86) | 0.45 (0.39 - 0.52) | 0.343 |
| SOFA | 0.68 (0.64-0.72) | 0.66 (0.62-0.69) | 0.74 (0.69 - 0.78) | 0.53 (0.46 - 0.59) | 0.73 (0.69 - 0.77) | 0.53 (0.47 - 0.59) | 8.5 |

**Table S5 sensitivity analyses of the association between PNI and all-cause mortality**

| **Variables** | **Time** |  | **Model** | |
| --- | --- | --- | --- | --- |
|  |  |  | **HR (95%CI)** | ***P*** |
| non-imputed dataset | D30 |  | 0.96(0.93, 0.98) | **<0.001** |
| Total ICU Patient Sample | D30 |  | 0.96(0.94, 0.98) | **<0.001** |
| Long-Term Mortality | D360 |  | 0.98 (0.96 ~ 0.99) | **0.037** |

Model adjusted for Age + Gender + Race + Insurance + SOFA + Charlson comorbidity index + APS III + Hemoglobin, Anion gap, INR, Total Bilirubin, and Sepsis
